# Cholesterol-Rich Lipid Rafts in the Cellular Membrane Play an Essential Role in Avian Reovirus Replication

**DOI:** 10.3389/fmicb.2020.597794

**Published:** 2020-11-02

**Authors:** Yuyang Wang, Yangyang Zhang, Chengcheng Zhang, Maozhi Hu, Qiuxiang Yan, Hongyan Zhao, Xiaorong Zhang, Yantao Wu

**Affiliations:** ^1^Jiangsu Co-innovation Center for Prevention and Control of Important Animal Infectious Disease and Zoonoses, College of Veterinary Medicine, Yangzhou University, Yangzhou, China; ^2^Testing Center, Yangzhou University, Yangzhou, China

**Keywords:** avian reovirus, cholesterol, lipid rafts, MβCD, virus replication

## Abstract

Cholesterol is an essential component of lipid rafts in cellular plasma membranes. Although lipid rafts have been reported to have several functions in multiple stages of the life cycles of many different enveloped viruses, the mechanisms by which non-enveloped viruses, which lack outer lipid membranes, infect host cells remain unclear. In this study, to investigate the dependence of non-enveloped avian reovirus (ARV) infection on the integrity of cholesterol-rich membrane rafts, methyl-β-cyclodextrin (MβCD) was used to deplete cellular membrane cholesterol at the ARV attachment, entry, and post-entry stages. Treatment with MβCD significantly inhibited ARV replication at both the entry and post-entry stages in a dose-dependent manner, but MβCD had a statistically insignificant effect when it was added at the attachment stage. Moreover, MβCD treatment markedly reduced syncytium formation, which occurs at a relatively late stage of the ARV life cycle and is involved in cell-cell transmission and release. Furthermore, the addition of exogenous cholesterol reversed the effects mentioned above. Colocalization data also showed that the ARV proteins σC, μNS, and p10 prefer to localize to cholesterol-rich lipid raft regions during ARV infection. Altogether, these results suggest that cellular cholesterol in lipid rafts plays a critical role in ARV replication.

## Introduction

Avian reovirus (ARV) is an important pathogen that causes viral arthritis syndrome and immunosuppression in domestic fowl, leading to considerable losses to the poultry industry (Jones and Kibenge, 1984; Neelima et al., 2003). ARV belongs to the *Orthoreovirus* genus of the *Reoviridae* family. ARV has a genome of 10 double-stranded RNA (dsRNA) segments enclosed in a double-protein capsid shell. Based on their electrophoretic mobilities, these segments are divided into L (large), M (medium), and S (small) classes, which encode the proteins designated lambda (λ), mu (μ), and sigma (σ), respectively. The ARV genome encodes eight structural proteins (λA, λB, λC, μA, μB, σA, σB, and σC) and four non-structural proteins (μNS, σNS, p10, and p17) (Benavente and Martinez-Costas, 2007).

ARV infection is initiated by the attachment of the outer capsid protein σC to cell surface receptors, and this binding event causes virions to enter cells through receptor-mediated endocytosis (Grande et al., 2002). Upon acidification of endosomes, virions are uncoated, and transcription-competent core particles are released into the cytosol to initiate viral gene expression (Duncan, 1996). Unlike the cellular inclusions in which most mammalian reoviruses form, the cellular globular viroplasm-like inclusions in which ARV morphogenesis occurs are not associated with microtubules. The non-structural protein μNS is the minimal viral factor that forms viroplasms (Touris-Otero et al., 2004b; Brandariz-Nunez et al., 2010). In the early stages of virus morphogenesis, μNS recruits σNS and λA to inclusions, generating sites of origin for viral replication and assembly (Touris-Otero et al., 2004a). After forming the mature virions, progeny viruses exit the infected host cell, causing cell lysis (Benavente and Martinez-Costas, 2007). Notably, the ARV, a fusogenic reovirus, is the only known example of a non-enveloped virus that can cause cell-cell fusion. Some data indicate that ARV-induced syncytium formation enhances virus transmission and release (Shmulevitz and Duncan, 2000; Salsman et al., 2005). The p10 protein probably plays a key role in virus release and dissemination. It is a member of the fusion-associated small transmembrane (FAST) protein family composed of proteins that induce cell-cell membrane fusion and syncytium formation (Ciechonska and Duncan, 2014; Duncan, 2019). Although some details of the ARV life cycle are well known, the underlying mechanism on how ARV is internalized into cells remains controversial, and its virion assembly and release are still poorly understood.

Lipid rafts are dynamic membrane microdomains highly enriched in cholesterol, sphingolipids, and specific raft-associated proteins and are present on the plasma membrane and the membranes of various intracellular organelles (Galbiati et al., 2001; Silvius, 2003). In these microdomains, sphingolipids, ganglioside GM_1_, and proteins are densely packed with high cholesterol concentrations that float-like rafts within the membrane bilayer (van Meer, 2002; Silvius, 2003). Lipid rafts have been demonstrated to regulate various biological processes, such as signal transduction, membrane transport, and protein trafficking (Anderson, 1998; Zhang et al., 1998; Brown and London, 2000).

In recent years, many enveloped viruses have been reported to associate with lipid rafts during virus entry, assembly, and/or budding because enveloped viruses are surrounded by lipid bilayers from the host plasma membrane (Suzuki and Suzuki, 2006; Lorizate and Krausslich, 2011). Cholesterol-rich regions usually serve as platforms for these viruses during the internalization and/or fusion processes in the entry stage (Helenius, 2018). This role of these cholesterol-rich regions has been demonstrated in studies of various enveloped viruses, such as human immunodeficiency virus (HIV), Newcastle disease virus (NDV), Semliki Forest virus (SFV), and avian sarcoma and leukosis virus (ASLV) (Ahn et al., 2002; Sáez-Cirión et al., 2002; Narayan et al., 2003; Martin et al., 2012). Moreover, the involvement of membrane lipid rafts has been observed in the intracellular assembly and release of several enveloped viruses, including influenza virus, HIV, NDV, and herpes simplex virus 1 (HSV-1) (Zhang et al., 2000; Laliberte et al., 2006; Ono, 2010; Wudiri and Nicola, 2017).

By comparison, studies characterizing the association of non-enveloped viruses with rafts are scarce. Traditionally, it was believed that events related to the entry of non-enveloped viruses proceed without membrane fusion and that these viruses are mainly released through cell lysis. However, there is increasing evidence that membrane lipid rafts are involved in the entry processes of non-enveloped viruses. Caveolae are a subset of lipid rafts that function in endocytosis, vesicular trafficking, cholesterol transport, and cell signaling (Nabi and Le, 2003; Quest et al., 2004). Simian virus 40 (SV40) binds to non-raft major histocompatibility complex (MHC) class I molecules, triggering its recruitment to caveolae located in lipid rafts. Then, caveolae-mediated endocytosis transports viral particles to the endoplasmic reticulum (ER) (Norkin et al., 2002). Caveolae or rafts have also been implicated in the entry of certain non-enveloped viruses, including coxsackievirus A9, echovirus 1, and mouse polyomavirus (Richterova et al., 2001; Marjomaki et al., 2002; Triantafilou and Triantafilou, 2003; Upla et al., 2004). At present, these microdomains’ function in the post-entry stage of the non-enveloped virus replication cycle is just beginning to be explored. Several studies have reported that cholesterol depletion inhibits infection with rotavirus, porcine rotavirus, and bluetongue virus beyond the viral entry stage (Cuadras and Greenberg, 2003; Bhattacharya and Roy, 2010; Dou et al., 2018). It has been mentioned that the rotavirus ER-transmembrane protein NSP4, which facilitates the budding of double-layered particles (DLPs) from viroplasms, associates with caveolae in plasma membrane rafts (Storey et al., 2007).

Although a previous study suggested that ARV might enter cells through a caveolin-1-mediated and dynamin-2-dependent endocytic pathway (Huang et al., 2011), cholesterol-rich rafts’ roles at the various stages of ARV infection are still elusive. The globular viroplasm formation and unusual fusogenic ability of ARV deserve attention. These functions indicate that ARV probably behaves similarly to enveloped viruses and interacts with intracellular and/or plasma membranes during virus morphogenesis and egress. In this study, quantitative analyses of attached virions, genome loads, progeny virus titers, and viroplasm and syncytium formation were conducted to evaluate cholesterol-rich lipid rafts’ involvement during ARV infection of Vero cells. Extraction of cholesterol by MβCD, replenishment of cholesterol, and microscopy-based colocalization analysis showed that cholesterol-rich lipid rafts are required for ARV replication at both the entry stage and the post-entry stage.

## Materials and Methods

### Cells and Virus

Vero cells were maintained in Dulbecco’s modified Eagle’s medium (DMEM; Gibco, Shanghai, China) supplemented with 10% fetal bovine serum (FBS) (Gibco) at 37°C with 5% CO_2_. The ARV strain GX/2010/1 was isolated by our laboratory and was propagated in chicken embryo fibroblast (CEF) cells (Meng et al., 2012; Duan et al., 2015; Niu et al., 2017, 2019).

### Reagents and Antibodies

Methyl-β-cyclodextrin (MβCD), cholesterol, 3-(4,5-dimethyl-2-thiazolyl)-2,5-diphenyl tetrazolium bromide (MTT), 4′,6-diamidino-2-phenylindole (DAPI) and dimethyl sulfoxide (DMSO) were purchased from Sigma-Aldrich (Shanghai, China). Filipin III was purchased from Cayman (Michigan, United States). A mouse monoclonal antibody against σC of ARV and polyclonal antibodies against μNS and p10 of ARV were prepared by our laboratory (Duan et al., 2015; Niu et al., 2019). A rabbit monoclonal antibody against Caveolin-1 was purchased from Cell Signaling Technology (Massachusetts, United States). A mouse monoclonal antibody against β-Actin and secondary antibodies, including fluorescein (FITC)-conjugated goat anti-mouse/rabbit IgG, horseradish peroxidase (HRP)-conjugated goat anti-mouse IgG, and cholera toxin subunit B (CTB) conjugated to Alexa Fluor^®^ 594 (CTB- Alexa Fluor 594), were purchased from Sigma-Aldrich. RIPA lysis buffer, PMSF, and SDS-PAGE loading buffer were purchased from Beyotime Biotechnology (Shanghai, China). The chemiluminescent substrate and DRAQ5 were purchased from Thermo Fisher Scientific (Shanghai, China).

### MTT Cell Viability Assay

Vero cells were seeded at a density of 5 × 10^4^ cells per well in 96-well plates containing DMEM supplemented with 10% FBS, and the cells were cultured at 37°C in 5% CO_2_. The cells were treated with MβCD at various concentrations (2.5, 5, 10, 15, and 20 mM) in serum-free DMEM for 2 h and then incubated in DMEM for 12 h. Then, a medium containing 30 μL of 2 mg/mL MTT was added to each well, and the plate was incubated at 37°C for 4 h. Finally, the supernatant was removed, 150 μL of DMSO was added to each well to solubilize the formazan, and the absorbance was read at a wavelength of 570 nm. A well containing pure DMSO was used as a blank. Mock-treated cells were used as controls, and the viability of these cells was set to 100%.

### Cell Membrane Cholesterol Staining and Measurement

Filipin III is a fluorescent antibiotic that binds specifically to cellular cholesterol and has a fluorescence spectrum similar to DAPI (Maxfield and Wustner, 2012). Thus, the decrease in membrane cholesterol levels induced by treatment with MβCD was verified by staining with filipin III. Vero cells were plated in 96-well plates and treated with various concentrations of MβCD (2.5, 5, 7.5, and 10 mM) for 45 min. After washing with cold phosphate-buffered saline (PBS) three times, the cells were fixed with 4% paraformaldehyde. Then, cell membrane cholesterol was labeled with 50 μg/mL filipin III (25 mg/mL stock in DMSO) in PBS for 2 h. High-content image analysis was used to quantify cell membrane cholesterol levels based on the fluorescence intensity of filipin III.

### Cholesterol Depletion at the Viral Attachment, Entry, and Post-entry Stages

To deplete cholesterol at the viral attachment stage, the cells were grown in monolayers in 6-well plates, mock-treated, or pretreated with the indicated concentrations of MβCD (5, 7.5, and 10 mM) for 45 min at 37°C and washed three times with cold PBS. The cells were inoculated with ARV at a multiplicity of infection (MOI) of 5 in ice-cold DMEM for 1 h at 4°C, which only allowed virus attachment. After the unbound virus was removed by extensive washing, the cell-associated virus was collected by freeze-thawing and quantified by virus titration.

To deplete cholesterol at the viral entry stage, the cells were grown in monolayers in 96-well plates for high-content image analysis. Cholesterol depletion was achieved by MβCD treatment at the indicated concentrations (2.5, 5, 7.5, and 10 mM) for 45 min, the medium containing MβCD was removed, and the cells were washed with PBS. The mock-treated or treated cells were infected with ARV at an MOI of 5 for 1 h at 37°C. Subsequently, the virus-containing medium was removed, and the cells were washed three times. Then, a fresh untreated medium supplemented with 2% FBS was added. The cells were incubated at 37°C until the mock-treated, virus-infected cells showed a complete cytopathic effect (CPE). The viroplasms were visualized by indirect immunofluorescence assay (IFA) after staining for the ARV-μNS protein, and ARV-positive cells were quantified by high-content analysis based on this staining. Moreover, cells grown in monolayers in 12-well plates were treated as described above for quantitative analyses of genome loads and virus titers. The level of ARV μNS gene expression in the cell lysates was measured by real-time PCR. Total progeny virus production in the cell lysates and supernatants was determined by calculating the TCID_50_ of CEF cells.

To deplete cholesterol at the viral post-entry stage, cells were infected with ARV at an MOI of 5 for 1 h at 37°C, and the remaining virus was removed by washing. The cells were mock-treated or treated with the indicated concentrations of MβCD (2.5, 5, 7.5, and 10 mM) for 45 min, washed and incubated in maintenance medium until the mock-treated, virus-infected cells showed complete CPE. The levels of the μNS protein, viral mRNA, and virus titers were quantified as described above.

To further study the effects of cholesterol depletion on the post-entry stage, which is involved in cell-to-cell virus spread, MβCD at a 5 mM concentration was administered after virus penetration but before cell lysis (at 1, 3, 6, and 9 hpi). Then, the cells were incubated at 37°C. ARV-infected cells not treated with MβCD showed extensive cell-cell fusion, and syncytium formation was evaluated by high-content image analysis. Furthermore, the total viral yield was measured by determining the TCID_50_ of CEF cells.

### Cholesterol Replenishment Experiment

Cholesterol replenishment was carried out to verify the effect of MβCD treatment on virus infection. At the viral entry and post-entry stages, MβCD (5 mM)-treated cells were incubated in DMEM containing 50 μM exogenous cholesterol for 1 h at 37°C. After washing, the cells were incubated at 37°C until the mock-treated, virus-infected cells showed complete CPE.

### Indirect Immunofluorescence Assay (IFA)

Cells were fixed with 4% formaldehyde for 15 min, permeabilized with 0.1% Triton X-100 in PBS for 5 min, and blocked with 5% BSA in PBS for 45 min. The cells were labeled with primary antibodies against μNS at a dilution of 1:200 in 1% BSA in PBS for 2 h and then incubated with a FITC-conjugated goat anti-mouse IgG secondary antibody at a dilution of 1:500 for 1 h. The nuclei were stained with DAPI, and the cells were washed with PBS for high-content image analysis.

### High-Content Image Analysis

The cells were screened and analyzed with the Operetta CLS^TM^ High-Content Analysis System (PerkinElmer, MA, United States). All the images were automatically analyzed using Harmony^®^ software with PhenoLOGIC (PerkinElmer, MA, United States) following the manufacturer’s instructions. To measure cell membrane cholesterol levels, images were acquired from 49 fields per well in the DAPI (filipin III shares a wavelength range with DAPI), brightfield, and digital phase control (DPC) channels 40 × objective. The fluorescence intensity of filipin III in the cell membrane was used to determine the cell membrane cholesterol content. For ARV μNS marker and syncytium analysis, images were collected from 25 fields per well in the FITC, DAPI, brightfield, and DPC channels with a 20× objective. Based on the histograms of the background fluorescence intensity from the mock-treated group, the populations presented with FITC intensities higher than 900 were identified as ARV-positive cells. For syncytium analysis, the cell nuclei were stained with DAPI, and multinuclear cells enclosed within a single plasma membrane (number of nuclei ≥ 3) were considered syncytia.

### Reverse Transcription and Real-Time PCR

Mock-treated and ARV-infected cells were lysed by a TRIzol reagent RNA kit (CWbio, Beijing, China), and total RNA was isolated according to the manufacturer’s instructions. For the SYBR Green-based real-time PCR assay, the total RNA concentration was measured by a NanoDrop 2000c spectrophotometer (Thermo, Massachusetts, United States), and 2 μg of RNA was reverse transcribed into cDNA in a 20 μL volume (TransGen, Beijing, China). Based on the complete genomic sequence of the ARV strain GX/2010/1 (GenBank accession numbers KJ476699-KJ476708), the forward and reverse PCR primer sequences used were as follows: ARV-μNS-F: 5′-CGTGTGGAAGCGTTAAACCA-3′, ARV-μNS-R: 5′-TCATCACGCTCGTTCAGGTC-3′. Absolute quantification of target genes was performed on an ABI Fast 7500 real-time PCR system (Applied Biosystems, Massachusetts, United States). Besides, a standard curve was generated from a 10-fold gradient dilution of μNS standard plasmids (pEGFP-μNS, prepared by our laboratory), and the μNS viral genome equivalent copies were calculated based on this standard curve.

### Virus Titration

The cell lysates harvested in the relevant experiments were serially diluted 10-fold in serum-free medium. The diluted viruses were inoculated in CEF cells to determine the 50% tissue culture infective dose (TCID_50_) by the Reed-Muench method.

### SDS-PAGE and Western Blot Analyses

SDS-PAGE and Western blot analyses were performed as previously described (Duan et al., 2015), and equal amounts of protein from each sample were loaded onto a gel. After transfer and blocking, the membranes were incubated with a primary polyclonal antibody against μNS (1:800 dilution) and a primary monoclonal antibody against β-actin (1:1,000 dilution) at room temperature for 4 h. The membranes were washed several times and incubated for 1 h at room temperature with an HRP-conjugated secondary anti-mouse antibody (1:5,000 dilution). The blots were developed using an enhanced chemiluminescence detection system (Tanon Imager, Shanghai, China).

### Confocal Microscopy

Colocalization of the σC or μNS protein with Caveolin-1 and the colocalization of p10 with cell membrane cholesterol or GM_1_ were observed by confocal microscopy. Cells on coverslips were processed as described for IFA. The coverslips were incubated with primary antibodies at a dilution of 1:200 in 1% BSA in PBS for 2 h and then incubated with secondary antibodies at a dilution of 1:500 for 1 h. Binding of CTB- Alexa Fluor 594 to ganglioside GM_1_ was used as an indicator of lipid rafts. Cell membrane cholesterol was labeled with filipin III. The nuclei were stained with DAPI or DRAQ5. Multichannel images of the samples were acquired with the LSM 880NLO instrument, and the extent of colocalization was analyzed quantitatively by ZEN 3.0 software (Zeiss, Jena, Germany).

### Statistical Analysis

All statistical tests were performed with IBM SPSS Statistics 19.0 (IBM, New York, United States). The data are expressed as the means ± SD. Significance was determined by one-way ANOVA (^∗^*P* < 0.05; ^∗∗^*P* < 0.01).

## Results

### Analysis of the Toxic Effects of MβCD and the Efficiency of Cell Membrane Cholesterol Removal

**FIGURE 1:**
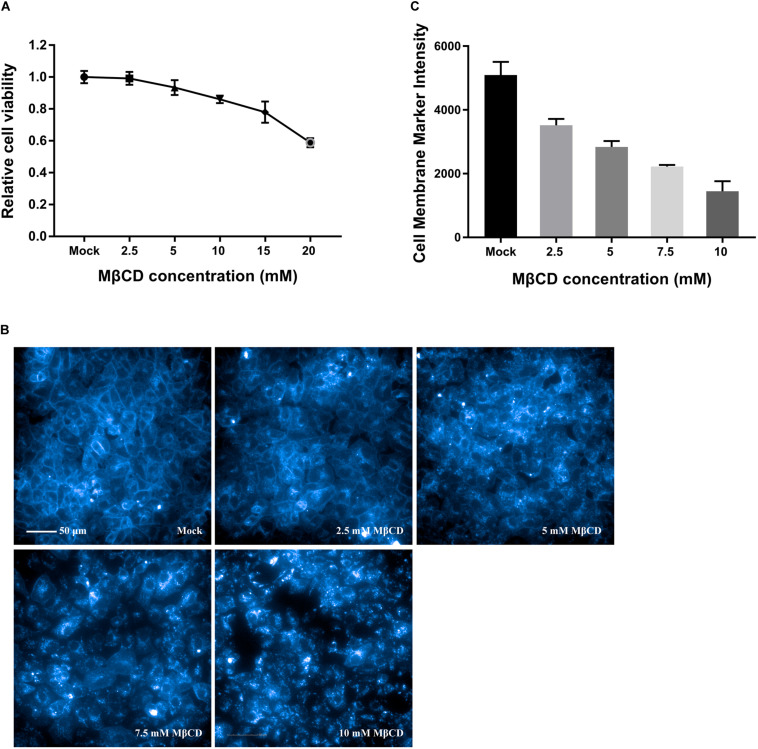
Cytotoxic effects of MβCD on Vero cells and the efficiency of cell membrane cholesterol removal by MβCD. **(A)** The toxic effect of MβCD on Vero cells was measured by the MTT assay. The error bars indicate the standard deviations of five independent experiments. **(B)** Vero cells were plated in 96-well plates and treated with various concentrations of MβCD (2.5, 5, 7.5, and 10 mM) for 45 min. Cell membrane cholesterol was labeled with 50 μg/mL filipin III, and images were acquired with the Operetta CLS^TM^ High-Content Analysis System. **(C)** The efficiency of cell membrane cholesterol removal by MβCD was measured by high-content analysis and determined by filipin III’s fluorescence intensity. The experiments were repeated twice, and the error bars indicate the standard deviations of the two independent experiments.

As shown by the MTT assay in Figure 1A, the cell monolayers remained intact, and more than 85% of the cells were viable after incubation with MβCD at concentrations of up to 10 mM. Cell membrane cholesterol was stained with filipin III, and the efficiency of cell membrane cholesterol removal by MβCD was measured by high-content image analysis. The results revealed a concentration-dependent reduction of cell membrane cholesterol (Figures 1B,C).

### Depletion of Cellular Cholesterol Does Not Block the Attachment of ARV

**FIGURE 2:**
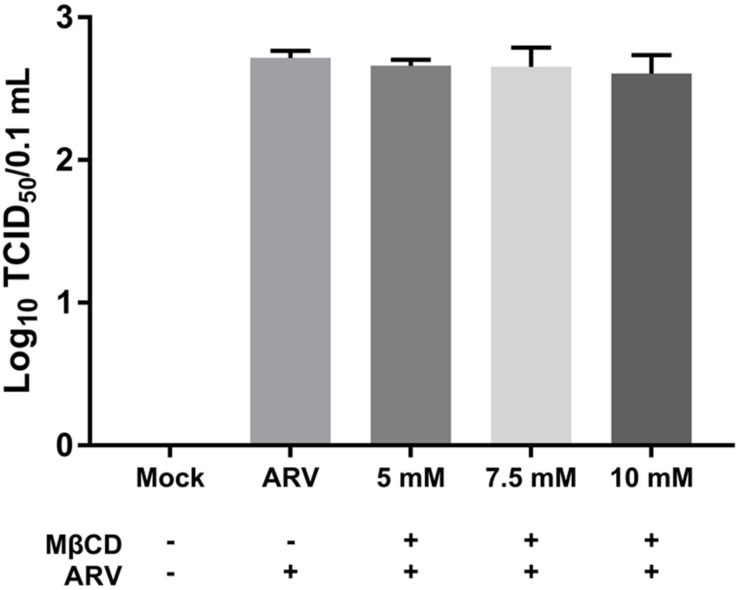
The depletion of cellular cholesterol did not block the attachment of ARV. Vero cells were mock-treated or pretreated with the indicated concentrations of MβCD (5, 7.5, and 10 mM) for 45 min at 37°C, and then, the virus binding assay was performed at 4°C. Viruses that were attached to cells were titrated, and the titers are expressed as the TCID_50_. The statistical analysis was based on three independent experiments, and the standard error is shown. Significance was determined by one-way ANOVA.

Equal amounts of ARV were added to the mock- or MβCD-treated cells, and the cells were incubated for 1 h at 4°C. This temperature only allowed viral attachment, and the process of virion internalization into the cells was inhibited. The viruses attached to the cells were titrated, and the titers are expressed as the TCID_50_. As shown by the viral load data in Figure 2, there was no significant difference between the MβCD-treated (5, 7.5, and 10 mM) and mock-treated ARV infection groups. The reduction in cellular cholesterol had a non-significant influence on the attachment of ARV to the target cells.

### Depletion of Cellular Cholesterol Inhibits ARV Replication at the Entry and Post-entry Stages in a Dose-Dependent Manner

**FIGURE 3:**
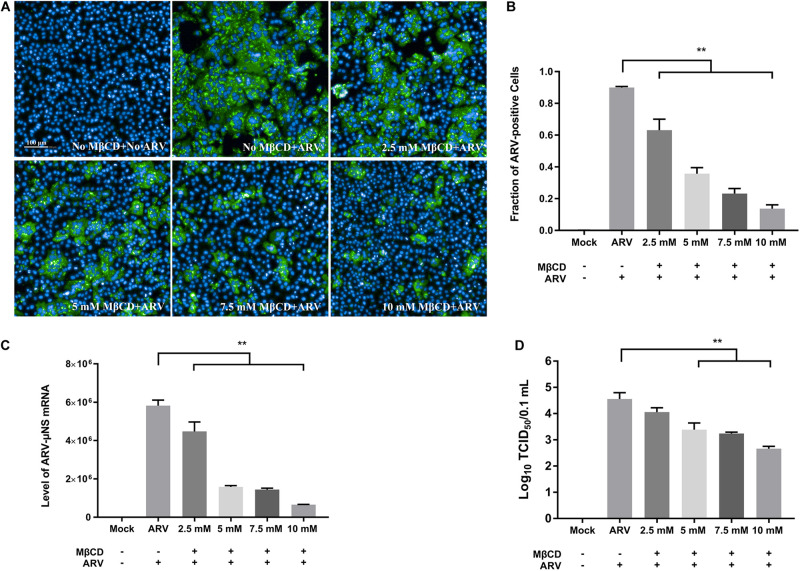
The effects of MβCD treatment before infection. The depletion of cellular cholesterol inhibits ARV replication at the entry stage in a dose-dependent manner. Before viral entry, cholesterol depletion was performed with MβCD pre-treatment at the indicated concentrations (2.5, 5, 7.5, and 10 mM). Mock-treated or treated cells were infected with ARV at an MOI of 5 for 1 h at 37°C. **(A)** Infected cells were analyzed by IFA at 18 hpi, and the labeled cells were imaged with the Operetta CLS^TM^ High-Content Analysis System. The fluorescence intensities of the μNS protein (green) and DAPI (blue) are shown. **(B)** The fraction of ARV-positive cells was measured by high-content analysis based on the fluorescence of the μNS proteins. **(C)** The mRNA expression levels of the ARV-μNS gene were determined by real-time PCR. **(D)** The number of infectious virus particles was quantified by TCID_50_ analysis. The statistical analysis was based on three independent experiments, and the standard error is shown. Significance was determined by one-way ANOVA (**P < 0.01).

**FIGURE 4:**
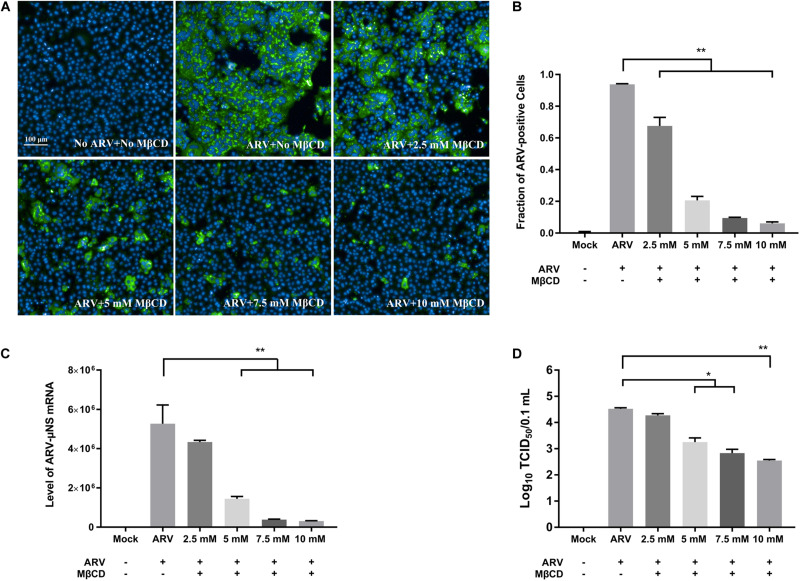
The effects of MβCD treatment after infection. The depletion of cellular cholesterol inhibits ARV replication at the post-entry stage in a dose-dependent manner. After the cells were infected with ARV at an MOI of 5 for 1 h at 37°C, cholesterol depletion was performed with MβCD treatment at the indicated concentrations (2.5, 5, 7.5, and 10 mM). **(A)** The infected cells were analyzed by IFA at 18 hpi, and labeled cells were imaged with the Operetta CLS^TM^ High-Content Analysis System. The fluorescence intensities of the μNS protein (green) and DAPI (blue) are shown. **(B)** The fraction of ARV-positive cells was measured by high-content analysis based on the fluorescence of the μNS proteins. **(C)** The mRNA expression levels of the ARV-μNS gene were determined by real-time PCR. **(D)** The number of infectious virus particles was quantified by TCID_50_ analysis. The statistical analysis was based on three independent experiments. Significance was determined by one-way ANOVA (*P < 0.05; **P < 0.01).

To investigate the roles of cholesterol during ARV infection, different concentrations (2.5, 5, 7.5, and 10 mM) of MβCD were used to deplete cell membrane cholesterol at the viral entry and post-entry stages. The ARV μNS levels in cholesterol-depleted cells were compared to those in untreated cells by IFA and high-content analysis at 18 hpi. The effects of MβCD treatment before infection are shown in Figure 3, and the effects of MβCD treatment after infection are shown in Figure 4. The analyses revealed that MβCD treatment remarkably inhibited μNS expression and viroplasm formation in ARV-infected cells at both the entry and post-entry stages of the ARV replication cycle (Figures 3A, 4A). Besides, with increasing concentrations of MβCD, the percentage of ARV-positive cells was reduced in a dose-dependent manner (Figures 3B, 4B). Furthermore, cell lysates were harvested from all experimental groups at 18 hpi. The mRNA expression levels of the μNS gene in each group were determined by real-time PCR (the PCR efficiency of the standard curve was 99.08%, data not shown), and the results are shown in Figures 3C, 4C. The progeny virus titers in the ARV-infected cells from each group were determined by measuring the TCID_50_, and the results are shown in Figures 3D, 4D. The real-time PCR and virus titration data revealed that the effects of cholesterol depletion at the entry and post-entry stages were similar to those revealed by ARV μNS protein quantification. Briefly, depletion of cellular cholesterol inhibited ARV replication after the virus entry stage in a dose-dependent manner.

### Cellular Cholesterol Depletion Significantly Influences Syncytium Formation Induced by ARV at the Post-entry Stage

**FIGURE 5:**
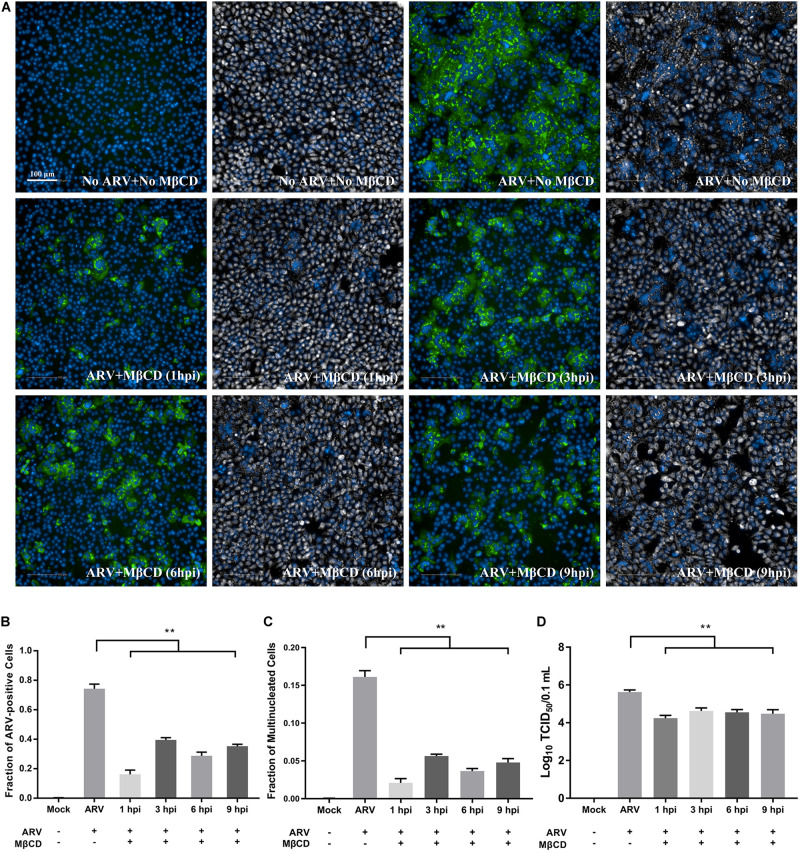
Cellular cholesterol depletion significantly influenced syncytium formation at the post-entry stage of ARV infection. **(A)** After the cells were infected with ARV at an MOI of 5 for 1 h at 37°C, cholesterol depletion was performed with 5 mM MβCD treatment at the indicated time points (1, 3, 6, and 9 hpi). The infected cells were analyzed by IFA at 18 hpi, and labeled cells were imaged with the Operetta CLS^TM^ High-Content Analysis System. The fluorescence intensities of the μNS protein (green) and DAPI (blue) are shown. The same fields in the DPC (gray) and DAPI (blue) channels are shown close to the fluorescence images. **(B)** The fraction of ARV-positive cells was measured by high-content analysis based on the fluorescence of the μNS proteins. **(C)** The fraction of multinucleated cells was measured with high-content analysis. **(D)** The number of infectious virus particles was quantified by TCID_50_ analysis. The statistical analysis was based on three independent experiments. Significance was determined by one-way ANOVA (**P < 0.01).

As shown in Supplementary Figure 1, Vero cells infected with ARV exhibited extensive cell-cell membrane fusion and syncytium formation, and a process that is involved in cell-to-cell virus spread and infectious virus release. To further assess cholesterol dependence in the post-entry stage of the ARV replication cycle, especially of ARV-induced membrane fusion prior to cell lysis, ARV-infected cells were treated with 5 mM MβCD at different time points. High-content image analysis indicated that ARV-induced syncytium formation was effectively reduced by cholesterol depletion by MβCD during the viral post-entry stage (Figures 5A,C). Additionally, the percentage of ARV-positive cells and the yield of ARV progeny in the MβCD-treated groups were lower than those in the mock-treated group (Figures 5B,D).

### Replenishment of Cholesterol Reverses the Inhibitory Effects of MβCD Treatment on ARV Infection

**FIGURE 6:**
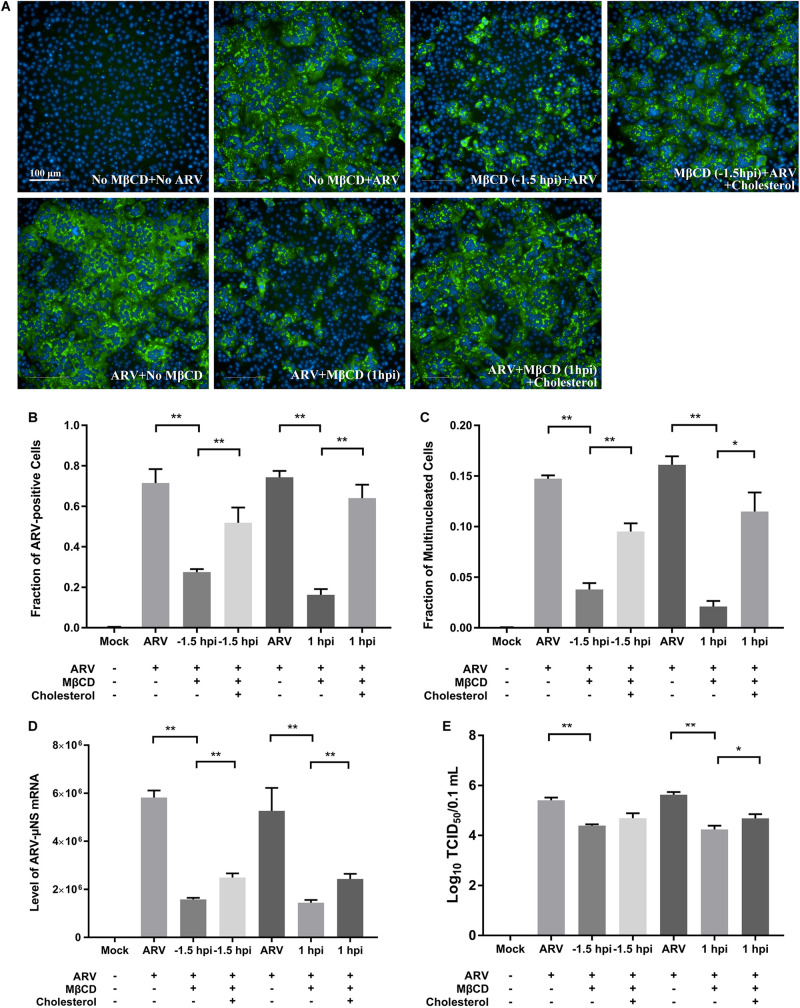
The replenishment of cholesterol reversed the inhibitory effects of MβCD treatment on ARV infection. Cholesterol replenishment was carried out at the viral entry or post-entry stages. MβCD (5 mM)-treated cells were incubated in DMEM containing 50 μM exogenous cholesterol for 1 h at 37°C. **(A)** The infected cells were analyzed by IFA at 18 hpi, and the labeled cells were imaged with the Operetta CLS^TM^ High-Content Analysis System. The fluorescence intensities of the μNS protein (green) and DAPI (blue) are shown. **(B)** The fraction of ARV-positive cells was measured by high-content analysis based on the fluorescence of the μNS proteins. **(C)** The fraction of multinucleated cells was measured with high-content analysis. **(D)** The mRNA expression levels of the ARV-μNS gene were determined by real-time PCR. **(E)** The number of infective virus particles was quantified by TCID_50_ analysis. The statistical analysis was based on three independent experiments. Significance was determined by one-way ANOVA (*P < 0.05; **P < 0.01).

**FIGURE 7:**
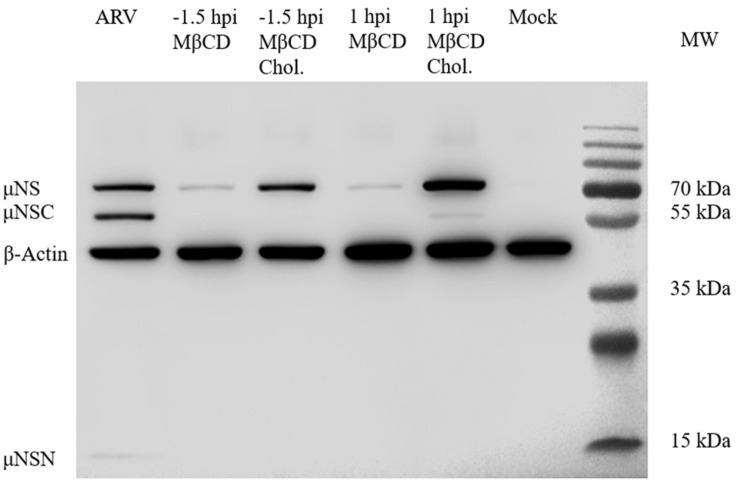
Western blot analysis of the μNS protein in cholesterol-depleted or cholesterol-replenished cells infected with ARV. A primary antibody against μNS was used to detect ARV. β-Actin served as the protein loading control. In ARV-infected cells, the μNS protein was cleaved into μNSN (15 kDa) and μNSC (55 kDa) (the biological significance of this cleavage is still unknown).

After depleting cellular cholesterol by 5 mM MβCD before or after ARV infection, cellular cholesterol was replenished by adding exogenous cholesterol (50 μM). As Figure 6 shows, compared with the mock treatment, treatment with exogenous cholesterol increased the number of μNS-positive cells by approximately 34 and 64% at the ARV entry and post-entry stages, respectively, as determined by high-content analysis (Figures 6A,B). The syncytium formation was also recovered by approximately 39 and 58% (Figure 6C). ARV μNS mRNA levels significantly rebounded, and progeny virus titers also recovered (Figures 6D,E). The results of Western blot analysis were consistent with those of IFA (Figure 7). In summary, the replenishment of cholesterol significantly restored ARV infection inhibited by MβCD, and these results suggest that the inhibitory effects of MβCD treatment on ARV replication could be attributed to cholesterol depletion.

### Colocalization of σC With Caveolin-1 During ARV Endocytosis

**FIGURE 8:**
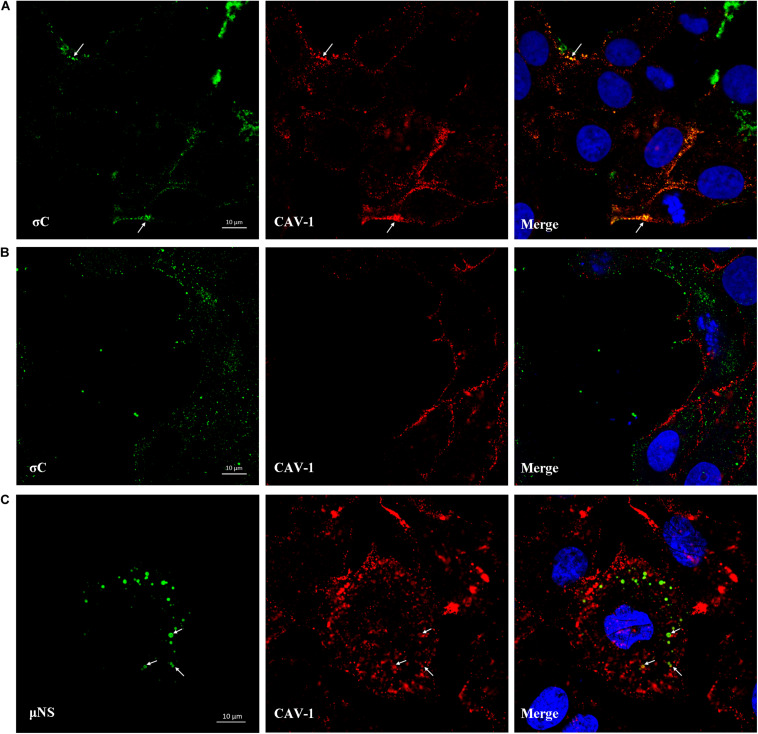
Colocalization of σC and μNS with Caveolin-1. **(A)** At 30 min post-infection at 37°C, the ARV-infected cells were immunostained with antibodies against σC (green) or Caveolin-1 (red). DAPI was used to stain the nuclei (blue). The white arrows indicate colocalization sites. **(B)** The ARV-infected cells at 30 min post-infection at 4°C were immunostained using antibodies against σC (green) or Caveolin-1 (red). DAPI was used to stain the nuclei (blue). σC did not colocalize with Caveolin-1. **(C)** Confocal images of ARV-infected cells immunostained using antibodies against μNS (green) or Caveolin-1 (red). DAPI was used to stain the nuclei (blue). The fluorescence signal of μNS (green) was presented as circular vesicles in the cytoplasm at 8 hpi, and some fluorescent vesicles seemed to aggregate to form larger viroplasm-like inclusions. The white arrows indicate colocalization sites.

IFA combined with confocal microscopy was used to visualize the subcellular colocalization of σC protein with Caveolin-1 during the ARV entry stage. As shown in Figure 8A, colocalization of σC and Caveolin-1 was detected after cells were infected with ARV at an MOI of 5 for 30 min at 37°C. Conversely, when cells were infected with ARV at 4°C, which only allowed virus attachment, σC was not found to colocalize with Caveolin-1 (Figure 8B). Moreover, the extent of colocalization is presented quantitatively as an X/Y scatter plot in Supplementary Figures 2A,B. Compared with the large colocalizing pixels in quadrant 3 of cells infected with ARV at 37°C, there were few colocalizing pixels in quadrant 3 of cells infected with ARV at 4°C.

### Colocalization of μNS With Caveolin-1

As previously reported, ARV morphogenesis occurs exclusively within globular cytoplasmic inclusions called viral factories; μNS is the minimal viral factor required for these viral factories and generates sites of origin for viral replication and assembly (Touris-Otero et al., 2004b). In our study, the fluorescent μNS protein was initially presented as circular in the cytoplasm in the viral factory’s infancy. Some circular structures seemed to aggregate to form larger viroplasm-like inclusions. The fluorescence signal of μNS was found to colocalize with Caveolin-1 at 8 hpi, as shown in Figure 8C. The extent of colocalization is presented quantitatively as an X/Y scatter plot in Supplementary Figure 2C. The presence of abundant colocalizing pixels in quadrant 3 indicated that μNS colocalized with Caveolin-1.

### Colocalization of p10 With Ganglioside GM_1_ and Cellular Cholesterol

**FIGURE 9:**
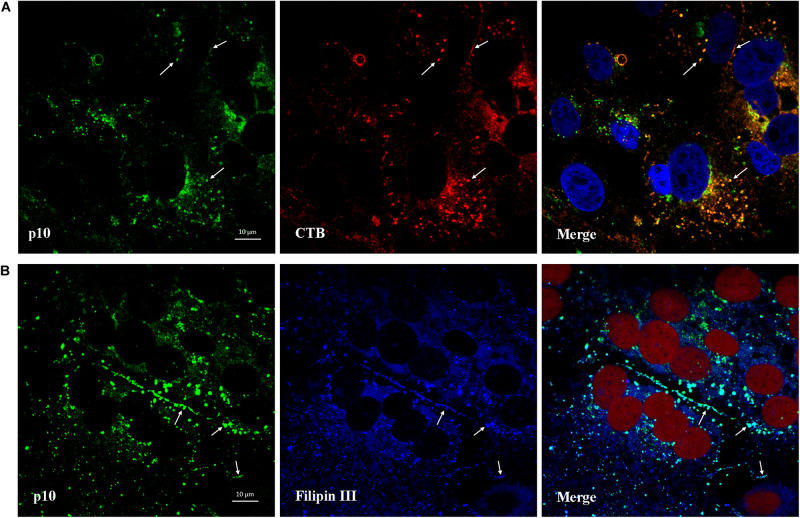
Colocalization of p10 with ganglioside GM_1_ and cellular cholesterol. **(A)** Confocal images of the ARV-infected cells costained with CTB (red) and antibodies against p10 (green) at 13 hpi. DAPI was used to stain the nuclei (blue). The white arrows indicate colocalization sites. **(B)** Confocal images of the ARV-infected cells costained with filipin III (blue) and antibodies against p10 (green) at 18 hpi. DRAQ5 was used to stain the nuclei (red). The white arrows indicate colocalization sites at the membrane boundaries.

CTB can be used as a marker for lipid rafts since it binds to the membrane microdomains via the pentasaccharide chain of ganglioside GM_1_. As shown in Figure 9A, after viroplasm-like inclusions formed, p10 was found to partially colocalize with ganglioside GM_1_ (CTB- Alexa Fluor 594) at 13 hpi. Furthermore, when obvious syncytia emerged in the ARV-infected cells at 18 hpi, p10 exhibited colocalization with filipin III-stained cholesterol on the cell membrane where cell-cell membrane fusion occurred (Figure 9B). The extent of colocalization is presented quantitatively as an X/Y scatter plot in Supplementary Figures 2D,E. The presence of abundant colocalizing pixels in quadrant 3 indicated that p10 colocalized with ganglioside GM_1_ and cellular cholesterol.

## Discussion

Cholesterol-rich lipid rafts are specific microdomains in the cell membrane enriched in cholesterol and sphingolipids (Brown and London, 2000). Since MβCD can extract cholesterol from the plasma membrane and intracellular membranes without affecting the membrane bilayer structure’s stability, depletion of cholesterol by MβCD is a commonly used method for evaluating the relationship between raft functions and virus replication (Ilangumaran and Hoessli, 1998). With this method, lipid rafts are shown to involve replicating various enveloped viruses and even those of some non-enveloped viruses (Ono and Freed, 2005; Suzuki and Suzuki, 2006; Lorizate and Krausslich, 2011). Although a previous study provided evidence that membrane cholesterol is essential for ARV endocytosis (Huang et al., 2011), whether membrane cholesterol affects other stages of the ARV life cycle, such as the ARV attachment or post-entry stages, remains to be determined. In this study, manipulation of cholesterol by MβCD treatment was used to evaluate the effect of a reduction in cellular cholesterol on the replication of ARV, including the processes of viral attachment, internalization, replication, syncytium formation, and egress. Here, ARV viroplasm and syncytium formation were visualized and quantified by the Operetta CLS^TM^ High-Content Analysis System. We first demonstrated that cellular cholesterol is a key determinant of ARV replication at both the entry and post-entry stages through real-time PCR and viral titration assays.

Virus particle attachment to the cell receptor-mediated by σC fibers is the first step of ARV entry, which triggers receptor-mediated endocytosis (Benavente and Martinez-Costas, 2007). To date, avian reovirus receptors have not been identified. In this study, depletion of cellular cholesterol did not block the attachment of ARV to the cell membrane at 4°C. At low temperatures, virus particle attachment proceeds without endocytosis. It has been speculated that ARV receptors would not localize to cholesterol-rich microdomains in the plasma membrane at the viral attachment stage. However, at 37°C, when the cells were treated with MβCD before or after virus entry, the depletion of cellular cholesterol inhibited ARV replication at the entry and post-entry stages in a dose-dependent manner. The replenishment of cholesterol significantly restored ARV infection that has been inhibited by MβCD.

Consequently, this finding indicated that cholesterol depletion affected the mechanisms involved in the virus’s internalization and one or more mechanisms that occur after virus entry. Besides, colocalization experiments revealed colocalization of σC and Caveolin-1 signals at 30 min post-infection at 37°C, but σC was not found to colocalize with Caveolin-1 at 30 min post-infection at 4°C. It has been shown that caveolins form hetero- and homo-oligomers that interact with cholesterol and other lipids in the lipid raft microdomain (Quest et al., 2004). Our data provided support for a previous study showing that ARV might utilize cholesterol- and caveolin-1-mediated and dynamin-2-dependent endocytosis pathway (Huang et al., 2011). Likely, the ARV receptors would not localize to lipid rafts areas in the plasma membrane at the viral binding stage. The virus particle-receptor complexes might move within the plasma membrane and subsequently localize to caveolae, followed by caveolae-dependent endocytosis.

ARV morphogenesis starts with the μNS-mediated formation of inclusions. Reovirus inclusions contain several types of filaments, viral proteins, ssRNAs, dsRNAs, and viral particles at various morphogenesis stages, and inclusions can be detected by light microscopy as early as 4 hpi (Fernandez de Castro et al., 2014). It was generally thought that reovirus inclusions do not contain membranes. However, a previous study showed that these inclusions are insoluble in Triton X-100-containing buffer and are not associated with microtubules (Touris-Otero et al., 2004a). The detergent-resistant property of μNS inclusions revealed that they might be closely related to lipid rafts.

Moreover, in recent years, Tenorio et al. (2018, 2019) used light and electron microscopy to demonstrate that reovirus inclusions are membrane-containing structures and that the reovirus σNS and μNS proteins remodel the ER to build neo-organelles for replication. Our results revealed that the depletion of cellular cholesterol inhibited ARV inclusion formation and replication at the post-entry stage in a dose-dependent manner. Besides, the μNS protein was found to colocalize with Caveolin-1 at the stage of μNS inclusion formation. Caveolin-1 is an essential structural component of cell surface caveolae, and it is also present in various intracellular organelle membranes, including the ER (Conrad et al., 1995). Based on these observations, we propose that μNS proteins initially interact with the raft regions of membranous tubules and vesicles derived from the peripheral ER’s extensive remodeling and utilize these membranous scaffolds to form globular viroplasms.

ARV is a rare case of a non-enveloped virus that causes cell-cell fusion. The non-structural protein p10 is synthesized intracellularly and transported to the plasma membrane. Fusion is triggered by p10 and is not linked to viral entry (Shmulevitz and Duncan, 2000). Recently, Kanai et al. demonstrated that reovirus FAST proteins enhance replication and pathogenicity of non-enveloped dsRNA viruses by inducing cell-cell fusion (Kanai et al., 2019). It has been reported that p10 induces cholesterol-dependent homo-multimerization to form fusion platforms in the plasma membrane (Key and Duncan, 2014). However, no studies have reported whether membrane cholesterol affects syncytium formation induced by ARV. Cholesterol depletion was performed at different time points after virus penetration in this study. The data showed that cholesterol depletion at the ARV post-entry stage markedly reduced syncytium formation. The total progeny viral load from the MβCD treatment groups was also reduced. Besides, analysis of the subcellular localization of p10 revealed colocalization with ganglioside GM_1_ and filipin III-stained cholesterol. These results indicated that cholesterol-rich lipid rafts participated in the intracellular synthesis of p10 and the cell-cell fusion induced by ARV infection. Thus, we conclude that non-structural p10 protein accumulated in the cholesterol-rich membrane microdomains of lipid bilayers after ARV infection and assembled multimerically to trigger the fusion process.

In conclusion, this study suggests that cellular cholesterol in lipid rafts is critical for ARV infection during the entry and post-entry stages rather than during virus attachment to target cells. Further study needs to be carried out to identify the potential sites of ARV-raft interactions and provide guidelines to develop novel antiviral strategies.

## Data Availability Statement

All datasets generated for this study are included in the article/Supplementary Material, further inquiries can be directed to the corresponding author.

## Author Contributions

YWa and YWu designed the study. YWa performed the research, analyzed most of the data, and wrote the manuscript. YZ, QY, and HZ carried out additional analyses. CZ, XZ, MH, and YWu contributed to refining the ideas and finalizing this manuscript. All the authors reviewed the manuscript.

## Conflict of Interest

The authors declare that the research was conducted in the absence of any commercial or financial relationships that could be construed as a potential conflict of interest.

## Supplementary Material

Supplementary Figure 1 Vero cells infected with ARV exhibited extensive cell-cell membrane fusion and multinucleated cell syncytia formation (in red circle). The left image was acquired in the brightfield channel, and the right merged image was acquired from the same field of view in the DAPI (blue) and DPC (gray) channels.

Supplementary Figure 2 X/Y scatter plot of quantitative colocalization analysis. The pixel intensities of two channels are plotted against one another in the diagram, and each pixel pair with the same X/Y image coordinates is displayed as a point. The frequency with which pixels of a certain brightness occur is visualized using a color palette displayed at the bottom of the diagram. The relative value range lies between 0 and 255. The vertical and horizontal axes show the gray value range that applies to the relevant channel. The diagram is overlaid with two lines that automatically subdivide it into 4 quadrants, numbered from 1 to 4. The quadrants have the following meanings: 1, non-colocalizing pixels from channel 1; 2, non-colocalizing pixels from channel 2; 3, colocalizing pixels; 4, background. **(A)** The X/Y scatter plot of Figure 8A. **(B)** The X/Y scatter plot of Figure 8B. **(C)** The X/Y scatter plot of Figure 8C. **(D)** The X/Y scatter plot of Figure 9A. **(E)** The X/Y scatter plot of Figure 9B.
