# Supplementary material for: Cholesterol-Rich Lipid Rafts in the Cellular Membrane Play an Essential Role in Avian Reovirus Replication
Source: Front Microbiol. 2020 Nov 2;11:597794. doi: 10.3389/fmicb.2020.597794 (PMC7667042; doi:10.3389/fmicb.2020.597794)
Supplement: Supplementary file 1 [file Data_Sheet_1.docx]

Supplementary Material

1. **Supplementary Figures**

**Supplementary Fig.1.** Vero cells infected with ARV exhibited extensive cell-cell membrane fusion and multinucleated cell syncytia formation (in red circle). The left image was acquired in the brightfield channel, and the right merged image was acquired from the same field of view in the DAPI (blue) and DPC (gray) channels.

**Supplementary Fig. 2.** X/Y scatter plot of quantitative colocalization analysis. The pixel intensities of two channels are plotted against one another in the diagram, and each pixel pair with the same X/Y image coordinates is displayed as a point. The frequency with which pixels of a certain brightness occur is visualized using a color palette displayed at the bottom of the diagram. The relative value range lies between 0-255. The vertical and horizontal axes show the gray value range that applies to the relevant channel. The diagram is overlaid with two lines that automatically subdivide it into 4 quadrants, numbered from 1-4. The quadrants have the following meanings: 1,non-colocalizing pixels from channel 1; 2,non-colocalizing pixels from channel 2; 3,colocalizing pixels; 4, background. (A) The X/Y scatter plot of Fig. 8A. (B) The X/Y scatter plot of Fig. 8B. (C) The X/Y scatter plot of Fig. 8C. (D) The X/Y scatter plot of Fig. 9A. (E) The X/Y scatter plot of Fig. 9B.
